# The Isolation and Full-Length Transcriptome Sequencing of a Novel Nidovirus and Response of Its Infection in Japanese Flounder (*Paralichthys olivaceus*)

**DOI:** 10.3390/v14061216

**Published:** 2022-06-02

**Authors:** Chunguang Gong, Yitong Zhang, Guixing Wang, Yufeng Liu, Zhongwei He, Yuqin Ren, Wei Cao, Haitao Zhao, Yuhao Xu, Yufen Wang, Jilun Hou

**Affiliations:** 1 Hebei Key Laboratory of the Bohai Sea Fish Germplasm Resources Conservation and Utilization, Beidaihe Central Experiment Station, Chinese Academy of Fishery Sciences, Qinhuangdao 066100, China; gongcg2005@163.com (C.G.); zhangyt@bces.ac.cn (Y.Z.); wanggx@bces.ac.cn (G.W.); liuyf@bces.ac.cn (Y.L.); hezw@bces.ac.cn (Z.H.); renyq@bces.ac.cn (Y.R.); caow@bces.ac.cn (W.C.); wangyf@bces.ac.cn (Y.W.); 2 Ocean College, Agricultural University of Hebei, Qinhuangdao 066000, China; xyh062315@126.com; 3 Hebei Ocean and Fisheries Science Research Institute, Qinhuangdao 066000, China; ninan-tao@163.com

**Keywords:** marine fish, viral genome, metabolism, alternative splicing, isoform sequencing

## Abstract

A novel nidovirus, CSBV Bces-Po19, was isolated from the marine fish, Japanese flounder (*Paralichthys olivaceus*). The viral genome was 26,597 nucleotides long and shared 98.62% nucleotide identity with CSBV WHQSR4345. PacBio Sequel and Illumina sequencing were used to perform full-length transcriptome sequencing on CSBV Bces-Po19-sensitive (S) and -resistant (R) Japanese flounder. The results of negative staining revealed bacilliform and spherical virions. There were in total 1444 different genes between CSBV Bces-Po19 S and R groups, with 935 being up-regulated and 513 being down-regulated. Metabolism-, immune-, and RNA-related pathways were significantly enriched. Furthermore, CSBV Bces-Po19 infection induced alternative splicing (AS) events in Japanese flounder; the S group had a higher numbers of AS events (12,352) than the R group (11,452). The number of long non-coding RNA (lncRNA) in the S group, on the other hand, was significantly lower than in the R group. In addition to providing valuable information that sheds more light on CSBV Bces-Po19 infection, these research findings provide further clues for CSBV Bces-Po19 prevention and treatment.

## 1. Introduction

The International Committee on Taxonomy of Viruses (ICTV) formally recognized the order Nidovirales in 1996, with four families: *Arteriviridae*, *Coronaviridae*, *Mesoniviridae*, and *Roniviridae* [1]. According to the later published taxonomy by ICTV in 2019 [2], there are 14 families in this order. Nidoviruses, a wide and varied category of positive-stranded RNA viruses, may infect hosts ranging from vertebrates to invertebrate animals, causing global outbreaks. One of the most notable examples is severe acute respiratory syndrome coronavirus 2 (SARS-CoV-2) virus, which belongs to the order *Nidovirales* and the family *Coronaviridae*. The SARS-CoV-2 virus triggered the breakout of the coronavirus disease 2019 (COVID-19) pandemic, which is yet ongoing. There have been few reports of nidoviruses in aquatic species, which are mostly confined to the subfamily *Piscanivirinae* and the family *Tobaniviridae* [3]. The *Piscanivirinae* subfamily is comprised of two genera, namely *Bafinivirus* and *Oncotshavirus*. White bream virus isolate DF24/00 (WBV DF24/00), the first fish nidovirus, was isolated in 2001 from white bream (*Blicca bjoerkna)* in Germany [4]. Other reported fish virus species in the subfamily *Piscanivirinae* include fathead minnow nidovirus (FHMNV) [5], chinook salmon bafinivirus isolate NIDO (CSBV NIDO) [6], yellow catfish bafinivirus (YCBV) [7], crucian carp nidovirus isolate HB93 (CCNV HB93) [1], chinook salmon bafinivirus isolate WHQSR4345 (CSBV WHQSR4345) [8], Atlantic salmon bafinivirus isolate VT01292015-09 (ASBV VT 01292015-09) [7], and chinook salmon bafinivirus isolate Cefas-W054 (CSBV Cefas-W054) [3]. These nidoviruses were only isolated from freshwater or anadromous migratory fish species. No nidovirus isolation from marine fish has been reported yet. 

Studies on virus–host interactions at various levels are critical for gaining a full understanding of virus properties. To understand the molecular processes of virus infection and possible locations of intervention, a thorough investigation of gene expression following virus infection at the entire genome scale is required. High-throughput sequencing is a precise method for investigating transcriptome data on fish immune to infection with bacteria, viruses, or parasites [9,10,11]. However, these investigations employed next-generation sequencing (NGS) technology, which has short sequencing run lengths, limiting the capacity to quantify transcript abundance [12]. The third-generation sequencing technology overcomes the limitations of NGS by enabling single-molecule, real-time isoform sequencing (Iso-Seq) with long read lengths, consistent coverage, and high accuracy. As a result, third-generation sequencing technology is more successful and effective in capturing the whole transcriptome [13]. 

Japanese flounder (*Paralichthys olivaceus*) is widely distributed along the Korean Peninsula, Japan, and China. In China, it is a commercially significant marine culture fish. The recent expansion of the fish culture business has resulted in several illnesses that have resulted in significant losses in the Japanese flounder population. The diseases induced by viral infection are becoming a major threat to the Japanese flounder culture [14]. 

This study reports the isolation of novel fish nidovirus CSBV Bces-Po19 from the marine fish Japanese flounder. The genetic and ultrastructural characterization of CSBV Bces-Po19 was performed. Third-generation sequencing technology was employed to generate full-length reference transcriptomes for Japanese flounder head kidney tissue, which was susceptible or resistant to CSBV Bces-Po19. A transcriptional scale comparison of the gene expression profiles of these samples was then carried out using NGS RNA-Seq. The findings of this study add to the knowledge of fish nidovirus, provide insight into the molecular mechanism of the host response to CSBV Bces-Po19 infection, and reveal CSBV Bces-Po19 prevention and therapy.

## 2. Materials and Methods

### 2.1. Fish Sampling and Virus Isolation

Japanese flounder were cultured at the Laoting culture base of Beidaihe Central Experimental Station, Chinese Academy of Fishery Sciences. During the spring of 2019, the fish were cultured at a water temperature of 13 to 14 °C and with a salinity of 30 to 32‰. A disease outbreak occurred, with a mortality rate ranging from 70 to 80%. Conventional antibiotic treatment did not affect the disease. Therefore, ten diseased Japanese flounders, 30–35 cm in length, were sampled. The spleen, kidney, liver, and gonad tissues were removed from each fish, pooled, and stored at −80 °C. 

The pooled tissues were homogenized in sterile PBS (1:10 weight–volume ratio) and centrifuged at 1000× *g* for 10 min. The supernatant was filtered through a 0.22 μm filter and inoculated onto a monolayer of epithelioma papulosum cyprinid (EPC), Chinook salmon (*Oncorhynchus tshawytscha*) embryonic tissue (CHSE), or Japanese flounder brain (JFB) cells in 6 well plates at 1:10 dilutions and was incubated at 17 °C in L15 with 5% fetal bovine serum for 20 days. The infected JFB cells and supernatant mixture were lysed for 3 cycles of freeze-thaw at −80 °C. Cell debris and contaminants were removed by centrifuging at 1807× *g* for 20 min at 4 °C. The purification of virions was performed using ultracentrifuge (himac CP70MX) according to the protocol described by Gao et al. [15].

### 2.2. Virus Genome Sequencing

To narrow down the range of candidate viruses, the RNA of virus-infected JFB cells was extracted using RNA simple total RNA kit (TIANGEN, Beijing, China), and cDNA was synthesized by PrimeScript RT reagent Kit with gDNA Eraser (Takara, Tokyo, Japan). The RNA was then used for small RNA library construction, siRNA sequencing, and analysis according to the protocol described by Wu et al. [16]. In particular, the siRNA faction between 18 to 26 nt in length was collected by acrylamide gel separation, and the selected siRNA was aligned to the reference virus genomes collected from the *GenBank Virus RefSeq* database using Bowtie software and then assembled into contigs by the SPAdes and Velvet software. After assembled contigs, annotation was conducted with five databases (*Host Genome, NCBI Nr, NCBI Nt, Virus RefSeq Nucletide,* and *Virus RefSeq Protein*).

To obtain the whole genome sequence of the isolated virus, the RNA of purified virions was extracted, and cDNA was synthesized. After quality checking, the sequencing library was constructed and clustered following the manufacturer’s recommended protocols. The library was sequenced on an Illumina NovaSeq 6000 platform and 150 bp paired-end reads were generated. The raw reads were trimmed using Trimmomatic [17], and the trimmed reads were aligned to the CSBV WHQSR4345 virus genome sequence (NCBI accession no. MG600027) using HISAT 2.1.0, and finally, the aligned sequences were assembled with MEGAHIT [18] to obtain the whole virus genome sequence.

### 2.3. Phylogenetic Analysis

The full genome sequence of the isolated virus in this work, as well as additional ten nidovirus sequences retrieved from NCBI, were utilized for phylogenetic analysis. The sequences were aligned, and phylogenetic relationships were inferred using the neighbor-joining method. The phylogenetic tree was constructed with 1000 bootstrap replications of the Tamura–Nei model using MEGA X [19].

### 2.4. RT-PCR Detection of Virus

For the detection of CSBV Bces-Po19, each reaction volume (50 μL) contained 200 ng of cDNA, 25 μL of 2×PrimeSTAR Max Premix (Takara, Tokyo, Japan), 300 nM of each primer, and ddH_2_O. The cycling conditions were as follows: 98 °C for 3 min; followed by 35 cycles of 10 s at 98 °C, 5 s at 55 °C, and 5 s at 72 °C on a GeneAmp PCR system 9700 (Applied Biosystem, Foster City, CA, USA). The primer sequences were listed in Table S1.

### 2.5. Histology and Transmission Electron Microscopy (TEM)

The gills, liver, spleen, kidney, heart, and stomach of diseased fish were sampled and fixed in Bouin’s solution for 24 h before being rinsed with 70% alcohol and kept at room temperature. The samples were dehydrated in ethanol serial dilutions, sectioned (5 μm thickness), and then stained with Harris’s hematoxylin and eosin. 

The liver, kidney, ovary, and spleen of diseased fish as well as inoculated JFB cells containing 70% cytopathic effects (CPEs) were processed for TEM (Hitachi HT7800, Hitachi, Tokyo, Japan) using the previously reported methodology [20]; the fixed tissues were then washed in 0.1 mol/L cacodylate buffer and embedded in resin, cured, and sectioned. The negative staining of pure virion was carried out in the manner described by Cano et al. [3]. Briefly, for all negatively stained samples, 4 μL of concentrated, purified virions were adsorbed to a glow-discharged carbon-coated grid. After a 1 min incubation, the virions were negatively stained with 1% phosphotungstic acid in water for 1 min and subsequently air-dried. All samples were then examined and respective sections photographed.

### 2.6. Iso-Seq Library Preparation, Sequencing, Assembly, and Annotation

We randomly selected 3 moribund fish that displayed symptoms (Sensitive (S)) and 3 fish that had no symptoms (Resistant (R)). Each fish’s head kidney was immediately frozen in liquid nitrogen for 48 h and kept at −80 °C until use. The TRIZOL Kit was used to extract RNA (Invitrogen, Carlsbad, CA, USA). To remove genomic DNA from extracted RNA, RNase-free DNase I was used. The quality of the extracted RNA was evaluated using an Agilent 2100 Bioanalyzer System (Agilent Technologies, Santa Clara, CA, USA). The presence of virus in all S and R samples was confirmed by PCR using specifically designed primers (Table S1) in the initial phase of the experiment (data not shown). To construct libraries for PacBio sequencing, quantified RNA from S1 to S3 was mixed in equal amounts in one pool, while RNA from R1 to R3 was mixed into another pool in similar amounts. The S and R libraries were prepared using the Clontech SMARTer PCR cDNA Synthesis Kit and the BluePippin Size Selection System technique as defined by Pacific Biosciences, following the Isoform Sequencing protocol (Iso-Seq) (PN 100-092-800-03). 

The sequence data obtained were processed with the SMRTlink 5.0 software. Circular Consensus Sequence (CCS) was created from subread BAM files with the following parameters: min_length 200, max_drop_fraction 0.8, no_polish TRUE, min_zscore −9999, min_passes 1, min_predicted_accuracy 0.8, and max_length 18,000. The output of CCS BAM files output was acquired as full-length reads and non-full-length reads using pbclassify.py script, ignore polyA false, and minSeqLength 200. The non-full-length and full-length fasta files were sent into the cluster step, which involved isoform-level clustering (ICE), followed by final arrow polishing, hq_quiver_min_accuracy 0.99; bin_by_primer, false; bin_size_kb, 1; qv_trim_5p, 100; and qv_trim_3p, 30. Using the Illumina RNA-Seq data and the LoRDEC program, further nucleotide mistakes in consensus reads were repaired [21]. Following that, consensus reads were aligned to the reference genome [22] using GMAP [23] with the parameters --no-chimeras -- cross-species --expand-offsets 1 -B 5 -K 50000 -f samse -n 1.

Unmapped transcripts and novel gene transcripts obtained from gene structure analysis were annotated as NT using BLAST (parameter: -outfmt 6; -evalue 1e-5; -max_target_seqs 10; -num_threads 4); the software Diamond BLASTX (parameter: --more-sensitive -k 10; -e 1e-5; -f 6; -p 4) was used to annotate to NR, KOG/COG, Swiss-Prot and KEGG; and the software of Hmmscan (parameter: -acc; --domtblout) was used in Pfam database analysis. 

### 2.7. Isoform Structure Analysis

TAPIS was used to analyze the gene structure of polished isoforms. The exon-intron structure of each transcript was predicted. Newly found loci and isoforms were identified by comparing them to the reference genome annotation, using the same criteria as for loci and isoform identification [24]. Alternative splicing (AS) events were analyzed using SUPPA [25]. The expression weight (Psi) of alternative splice based on transcript TPM values was calculated. Differential alternative splice of two conditions was performed using significance test of Psi. The dpsi value was adjusted using the Mann–Whitney U test method. The absolute dpsi value of 0.1 and *p*-value of 0.05 were set as the threshold for significantly differential alternative splice. The full-length transcript that was mapped on two or more loci in the reference genome with more than 99% alignment coverage and having the locus 100 kb apart from each other on the reference genome and a 10% alignment of each gene locus to the corresponding transcript was predicted as a fusion transcript [24]. 

### 2.8. Transcript Factor and LncRNA Analysis

The animal TFDB 2.0 database was used for transcript factor (TF) analysis [26]. The transcripts were evaluated for LncRNA prediction using PLEK (Predictor of Long Non-coding RNAs and Messenger RNAs based on an Improved K-mer Scheme) with default settings of -minlength 200 and CNCI (Coding–Non-Coding Index) with default parameters for the first stage. For the second stage, the remaining transcripts were evaluated using CPC (Coding Potential Calculator) with settings of e-value “1e-10”. Finally, the transcripts were searched against the Pfam database using the default settings -E 0.001 --domE 0.001. Transcripts projected to have coding potential by one or more of the four methods mentioned above were filtered out, and those lacking coding potential were chosen as a candidate set of lncRNAs.

### 2.9. RNA-Seq by Illumina Hiseq XTen

Total RNA from S1 to S3 and R1 to R3 were prepared separately for RNA-Seq. Briefly, the sequencing libraries were created with the NEBNext UltraTM RNA Library Prep Kit for Illumina (NEB, Ipswich, MA, USA) and then tested for quality with an Agilent Bioanalyzer 2100. Following library preparation, index-coded samples were clustered, and libraries were sequenced using an Illumina Hiseq XTen platform, yielding paired-end reads. The raw sequencing data presented in this paper were deposited in the Genome Sequence Archive in National Genomics Data Center, Beijing Institute of Genomics (China National Center for Bioinformation), Chinese Academy of Sciences, under accession number CRA003368 that are publicly accessible at https://bigd.big.ac.cn/gsa (Unpublished Data).

### 2.10. Differential Expression Analysis and Functional Enrichment

Raw reads were cleaned before being mapped to the Japanese flounder reference genome [22] using Hisat2 (v2.1.0) [27]. The number of reads mapped onto each gene was counted using HTSeq (v0.6.1) [28]. The FPKM of each gene was calculated using the length of the gene and the number of reads mapped to that gene. The DESeq R program was used to perform differential expression analysis of (1.18.0). DESeq determined that genes with an adjusted *p* < 0.05 were differentially expressed. 

GOseq (v1.10.0) was used to perform a gene ontology (GO) enrichment study of differentially expressed genes (DEGs) [29]. GO keywords with a corrected *p* < 0.05 were deemed significantly enriched. The statistical enrichment of DEGs in KEGG pathways was tested using KOBAS (v3.0) [30], which was used to test the statistical enrichment of DEGs in KEGG pathways. 

### 2.11. qRT-PCR Validation

qRT-PCR was used to confirm the RNA-Seq data. Ten DEGs with high levels of significance were selected for qRT-PCR analysis, with the 18S gene serving as a control (Table S1). The qRT-PCR was performed using SYBR Green and BIO-RAD CFX96 Real-Time PCR under the following conditions: 94 °C for 2 min, followed by 40 cycles at 94 °C for 30 s and 60 °C for 20 s, while the 2^−ΔΔCt^ method was used to analyze gene expression levels. 

## 3. Results

### 3.1. Pathology of Diseased Fish

**Figure 1:**
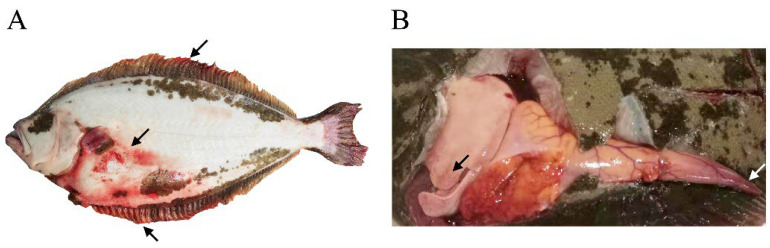
Clinical signs of CSBV Bces-Po19 infected Japanese flounder (*Paralichthys olivaceus*). The clinical signs observed in the moribund fish were partial body surface reddening, especially hemorrhages in fins and abdominal muscles. (**A**) The blind side of fish with CSBV Bces-Po19 disease; (**B**) visceral organs of the abdomen. Arrows indicate the hemorrhage positions.

**Figure 2:**
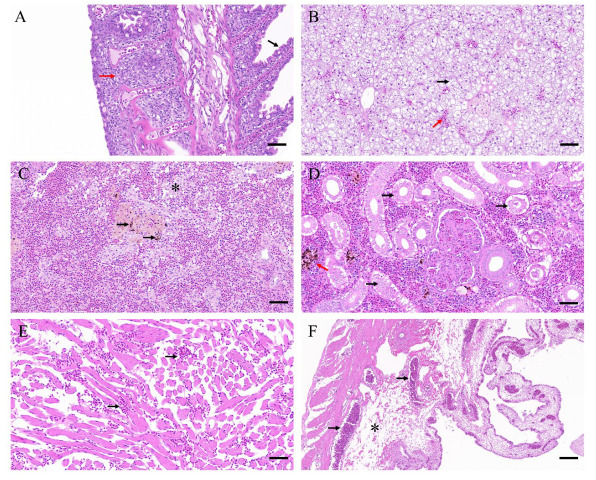
Histopathology of CSBV Bces-Po19 infected Japanese flounder (*Paralichthys olivaceus*). (**A**) Gill, with black arrow indicating the gill lamella with slight hyperplasia; red arrow indicates the gill lamella in stick shape with obvious hyperplasia. (**B**) Liver, with black arrow indicating the vesicular degeneration of hepatocytes; red arrow indicates sinus dilatation and blood cells extravasation. (**C**) Spleen, with black arrows indicating the hemolytic plaque; asterisk indicates vacuolated splenocytes. (**D**) Kidney, with black arrows indicating tubular epithelial cells necrosis; red arrow indicates renal stroma hemolysis. (**E**) Heart, with black arrows indicating the congestive tissue with erythrocytes. (**F**) Stomach, with black arrows indicating infiltrated hemocytes in the submucosa; asterisk indicates necrotic loose connective tissue of submucosa. Scale bars: 40 µm.

The moribund fish exhibited bleeding on their fins, muscles, liver, gonads, etc. (Figure 1). The infection caused varying degrees of gill lamella hyperplasia and lamellar fusion in the gills (Figure 2A). Pathologies such as vesicular degeneration of hepatocytes, sinus dilatation, and blood cells extravasation were observed in the liver (Figure 2B). The main pathological changes in the spleen were hemolytic plaque and vacuolated splenocytes together with elevated levels of neutrophils (Figure 2C). In the kidney, tubular epithelial cell necrosis and renal stroma hemolysis appeared (Figure 2D). The heart tissues were found congested due to viral infection (Figure 2E). The predominant pathogenic phenotypes in the stomach were infiltrating hemocytes and necrotic loose connective tissue of the submucosa (Figure 2F).

### 3.2. Virus Isolation and Genome Sequence Analysis

The infected EPC, CHSE, and JFB cells all demonstrated severe CPEs that resulted in large plaques of detached cells and the loss of the monolayer (Figure S1). The infected JFB cell short RNA sequencing yielded 16,476,179 clean reads, accounting for 97.24% of the total reads. After analysis, there were 12 contigs assigned to six candidate viruses. Among them, CSBV WHQSR4345 had the highest hit length rate of 96.97% (Table S3). Therefore, the genome of CSBV WHQSR4345 was used as a reference sequence in the following analysis.

**Figure 3:**
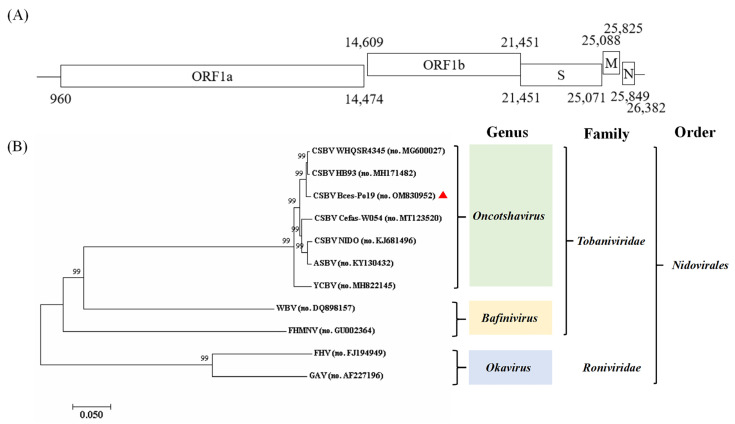
The genetic characteristics of CSBV Bces-Po19. (**A**) Structural organization of the CSBV Bces-Po19 genome. (**B**) Phylogenetic analysis of CSBV Bces-Po19 and other aquatic nidoviruses.

The isolated virus was named as CSBV Bces-Po19, and its full-length genome sequence (NCBI accession no.: OM830952) was 26,597 nt in length, including a 5′ UTR (959 nt) and five open reading frames (ORFs) that encode ORF1a (13,515 nt; 4504 aa), ORF1b (6843 nt; 2280 aa), spike glycoprotein (S; 3618 nt; 1205 aa), membrane protein (M; 738 nt; 245 aa), nucleocapsid protein (N; 534 nt; 177 aa), and a 3′ UTR (215 nt) (Figure 3A). 

To identify the genetic relationship between CSBV Bces-Po19 and other viruses belonging to the genus *Oncotshavirus,* both nucleotide and amino acid homology analysis were performed. Phylogenetic analysis of the viral genome placed the CSBV Bces-Po19 in the *Oncotshavirus* genus, within the *Tobaniviridae* family of *Nidovirales*, forming a clade with CSBV WHQSR4345 (NCBI accession No. MG600027) and CSBV HB93 (NCBI accession No. MH171482) (Figure 3B). 

The comparison of complete genome sequences in GenBank Blast searches showed that the CSBV Bces-Po19 isolate had a similarity of 93.17–98.62% (in nucleotides) to other aquatic nidoviruses isolates, with the highest percent identity to CSBV WHQSR4345. Nucleotide and amino acid sequence analyses of five ORFs are shown in Tables S4 and S5. Among the aquatic oncotshaviruses, CSBV WHQSR4345 has the highest identity; all other virus species have an ORF with less than 90% identity (both in nucleotides and amino acids).

### 3.3. RT-PCR Detection of Virus

**Figure 4:**
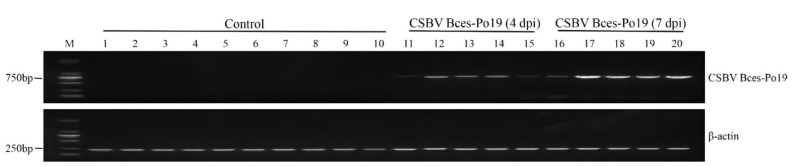
RT-PCR detection of CSBV Bces-Po19 in JFB cells. M, DL2000 DNA marker; Lanes 1–5, control JFB cells cultured for 4 days; Lanes 6–10, control JFB cells cultured for 7 days; Lanes 11–15, CSBV Bces-Po19-infected JFB cells for 4 days; Lanes 16–20, CSBV Bces-Po19-infected JFB cells for 7 days.

Using the designed CSBV Bces-Po19-specific primers, bands with 757 bp were detected in CSBV Bces-Po19-infected JFB cells, while no band was detected for the control cells. However, the internal reference gene β-actin was found in all samples in both control and infected cells (Figure 4). This result demonstrated that the CSBV Bces-Po19 virus could be detected using RT-PCR technology and specific primers. 

The primers used in this experiment were designed to amplify ORF1a of CSBV Bces-Po19. However, due to the close species relatedness, CSBV Bces-Po19 primer pairs could cross-react with CSBV WHQSR4345, CSBV HB93, and CSBV Cefas-W054. As such, the viral species should be initially screened by PCR and then further validated through whole-genome sequencing.

### 3.4. Ultrastructural Pathology and Characterization of Virion

**Figure 5:**
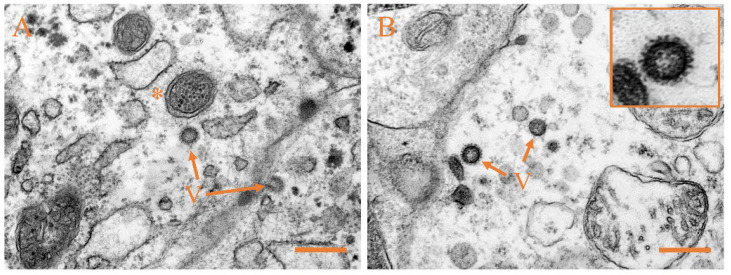

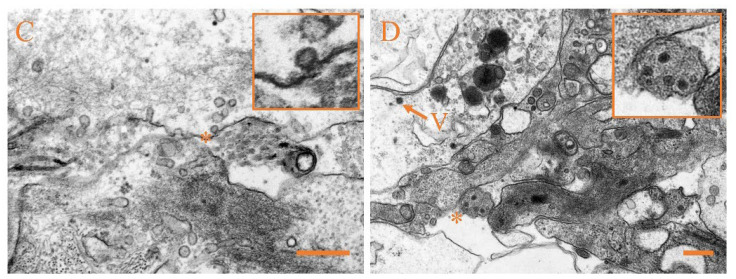
Transmission electron micrographs of liver, kidney, ovary, and spleen from CSBV Bces-Po19-infected Japanese flounder (*Paralichthys olivaceus*). (**A**) Liver, with arrows indicating the mature virions; asterisk indicates the mitochondrion with virion-like, granular osmiophilic substances. (**B**) Kidney, with arrows indicating the mature virions. (**C**) Ovary, with asterisk indicating viral budding from the infected cell membrane. (**D**) Spleen, with arrow indicating the mature virion; asterisk indicates the mitochondrion with virion-like, granular osmiophilic substances. Scale bars: 400 nm.

The ultrastructural examination was performed on the liver, kidney, ovary, and spleen of Japanese flounder infected with CSBV Bces-Po19. Cell necrosis was the most common pathogenic alteration in all four tissues. The spherical virions were visible, measuring 55–110 nm in diameter, and featured surface spikes that protruded 14–21 nm from the virion wrap (Figure 5A,B). The virion was detected in the process of budding out of an infected cell (Figure 5C). After viral infection, the mitochondrial structures were damaged, and granular osmiophilic substances such as spherical virions were found in the mitochondria (Figure 5A,D).

**Figure 6:**
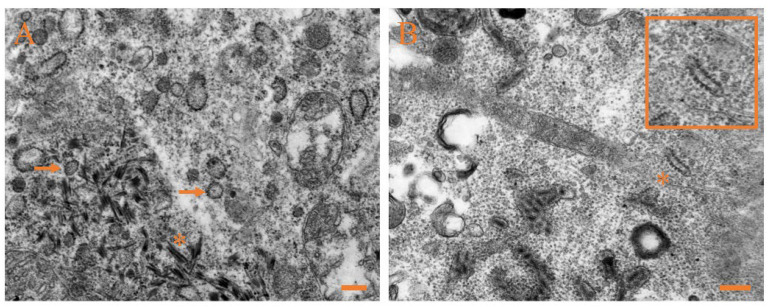

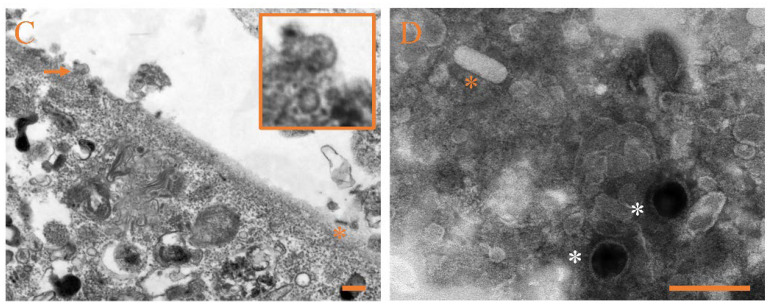
Transmission electron micrographs of JFB cells inoculated with CSBV Bces-Po19. (**A**) Arrows indicate the spherical virions; asterisk indicates viral-like tubular structure. (**B**) Asterisk indicates bacilliform virion. (**C**) Arrow indicates the spherical virion budding from the infected cell membrane; asterisk indicates bacilliform virion. (**D**) Transmission electron microscopy (negative staining) of purified virions from the JFB cell supernatant. Orange asterisks indicate bacilliform virions; white asterisks indicate spherical virions. Scale bars: 200 nm.

Pathological alterations in CSBV Bces-Po19-infected JFB cells included necrosis and extensively disrupted spherical and bacilliform virions (Figure 6A–C). In the early stages of infection, the cytoplasm was filled with viral-like tubular structures. (Figure 6A). The tubules were cylindrical, ranging in length from 80 to 215 nm and diameter from 15–20 nm. TEM analysis of pure virions (negative staining) from JFB cells revealed bacilliform viral particles of ≈50 nm in diameter and ≈160 nm in length as well as spherical viral particles of ≈100 nm in diameter (Figure 6D).

### 3.5. RNA-Seq Results

From the head kidney samples of CSBV Bces-Po19-sensitive and -resistant Japanese flounder, Illumina sequencing yielded 41,175,958 to 55,892,426 clean reads. The Japanese flounder reference genome was uniquely mapped to an average of 90.13% of clean reads (Table S2). FPKM value was calculated using the HTSeq program to assess relative abundance. Finally, 13,872 genes with FPKM > 1 were identified in six samples on average (Table S2).

### 3.6. Iso-Seq Results

The Iso-Seq on PacBio Sequel machine produced 23.31 and 24.30 G polymerase read bases for CSBV Bces-Po19 R and S samples, respectively (Table 1). After eliminating adaptors and reads with lengths less than 50 bp, the subreads bases for R and S were 22.43 and 23.62 G, respectively. Following filtering, 450,095 and 435,572 circular consensus (CCS) readings were retrieved. For samples, R and S, which comprised poly-A, 5′, and 3′ primers, the number of full-length non-chimeric reads was 386,725 and 383,604 (85.92% and 88.07% of total CCS reads), respectively. In subsequent analysis, polished consensus reads, 212,319 (R) and 183,681 (S), were utilized (Table 1).

The polished consensus reads were adjusted with Illumina RNA-Seq data to reduce further nucleotide mistakes in full-length sequencing reads. After correction, 463,495,705 (R) and 535,777,939 (S) nucleotides were retrieved, representing 99.86% and 99.84% of the total nucleotides before correction, respectively (Table S6). The N50 of samples R and S after the correction was found to be 2 bp and 9 bp shorter than before the correction, respectively; however, N90 for both samples R and S was just 1 bp shorter, demonstrating that Iso-Seq is extremely accurate. 

The polished consensus reads, after correction, were mapped to the reference genome of Japanese flounder, yielding a mapping rate of 86.84% (R) and 84.60% (S), respectively (Table S7). There were 45,901 (R) and 42,177 (S) isoforms found in total (Table S8). The majority of the isoforms discovered were novel isoforms of known genes (40,423 for R; 38,716 for S), where only 2641 and 2502 isoforms were previously detected. R and S groups shared 14,141 of these isoforms. However, the S group had many fewer isoforms than R group, particularly for novel isoforms of known genes and isoforms of novel genes.

### 3.7. Analysis of DEGs

**Figure 7:**
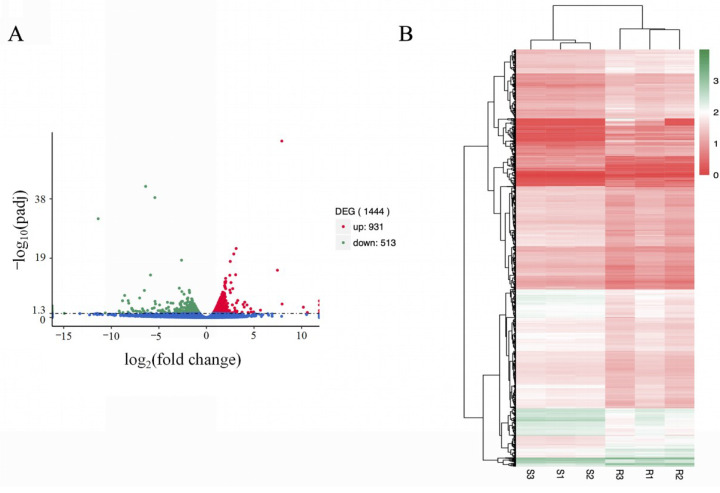
The differentially expressed genes (DEG) between CSBV Bces-Po19-sensitive and -resistant Japanese flounder (*Paralichthys olivaceus*). (**A**) Volcano map of DEGs. Each dot represents one gene, red dots represent the significantly up-regulated genes, and green dots represent the significantly down-regulated genes. Blue dots represent genes that are not significantly differentially expressed. (**B**) Heatmap analysis of the hierarchical clustering of DEGs. Different colors indicate differences in the expression level.

**Figure 8:**
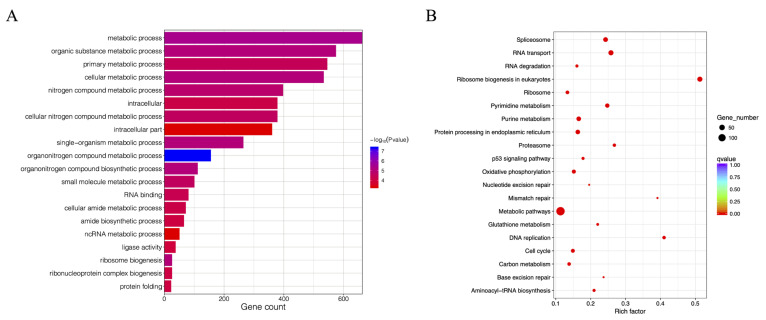
GO and KEGG enrichment of differentially expressed genes (DEG) between CSBV Bces-Po19-sensitive and -resistant Japanese flounder (*Paralichthys olivaceus*). (**A**) GO enrichment of DEGs. The names of the GO categories are listed along the *y*-axis; the degree of GO enrichment is represented by the −log_10_ (*p*-value). (**B**) KEGG enrichment of DEGs. The *x*-axis represents the gene ratio, which is a proportion of the DEGs out of the total genes in a KEGG term. *y*-axis is the gene function classification of KEGG. Different plot colors indicate different q values. Plot diameter represents the DEG numbers in a KEGG term.

DEG analysis was used to analyze the transcriptome profile of Japanese flounder in response to CSBV Bces-Po19. A comparison of the CSBV Bces-Po19 S and R transcriptomes revealed 1444 DEGs, 931 of which were highly up-regulated and 513 of which were significantly down-regulated (Figure 7A). A heatmap analysis of the hierarchical clustering was used to establish the profiles of the DEGs (Figure 7B). DEGs were subsequently examined for GO and KEGG functional enrichment to determine their primary molecular function during CSBV Bces-Po19 infection. DEGs were divided into three categories based on their GO functional enrichment analysis: biological process (65 level-3 subclasses), cellular component (16 level-3 subclasses), and molecular function (45 level-3 subclasses). Figure 8A shows the top 20 enriched GO keywords. 

A KEGG pathway analysis found that 129 pathways were implicated in the Japanese flounder’s reaction to CSBV Bces-Po19. Ribosome biogenesis in eukaryotes, metabolic pathways, RNA transport, spliceosomes, and pyrimidine metabolism are the top five enriched pathways (Figure 8B). Interestingly, 59 signaling pathways associated with metabolism, such as pyrimidine metabolism, purine metabolism, and glutathione metabolism, were enriched; they account for 45.94% of the total 129 pathways (Table S9). Eleven immune-related signaling pathways were found to be enriched, including the p53 signaling pathway, the phagosome, and the cytosolic DNA-sensing route (Table 2).

### 3.8. Detection of AS Events

**Figure 9:**
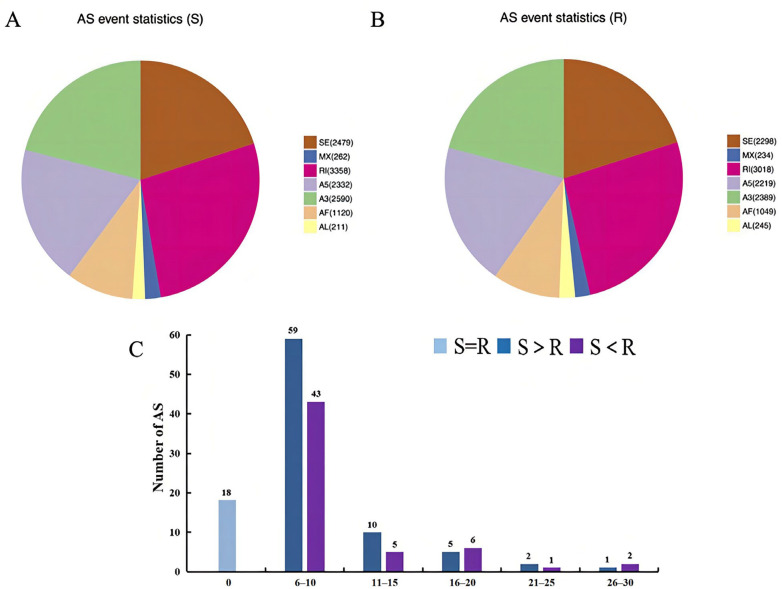
Alternative splicing (AS) analysis of the full-length transcriptome of CSBV Bces-Po19-sensitive and -resistant Japanese flounder (*Paralichthys olivaceus*). (**A**) AS events statistics of CSBV Bces-Po19-sensitive (S) group. (**B**) AS events statistics of CSBV Bces-Po19-resistant (R) group. (**C**) Comparison of the number of different AS in genes between CSBV Bces-Po19 S and R group.

SUPPA software was used to identify seven types of AS events: skipped exon (SE), mutually exclusive exon (MX), alternative 5′ splice site (A5), alternative 3′ splice site (A3), retained intron (RI), alternative first exon (AF), and alternative last exon (AF) (AL). There were 12,352 AS events in 10,743 of the total S group genes and 11,452 AS events in 12,989 of the total R group genes (Table 3). This suggests that CSBV Bces-Po19 infection may cause AS episodes in Japanese flounder. RI was the most common kind of AS in both the S and R groups, followed by A3, SE, and A5 (Figure 9A,B). 

A comparison of CSBV Bces-Po19 S and R groups revealed 152 genes with varying amounts of AS. In 57 of these 152 genes, the R group had more AS than the S group, while the S group had more AS than the R group in the other 77 genes (Figure 9C). The *tead3* gene had the most AS in the R group, with 52 AS, which is 25 higher than the S group. The highest number of AS was found in the S group in the *slco4a1* gene, which contained 57 AS, which is 21 more than the R group (Table 4).

**Figure 10:**
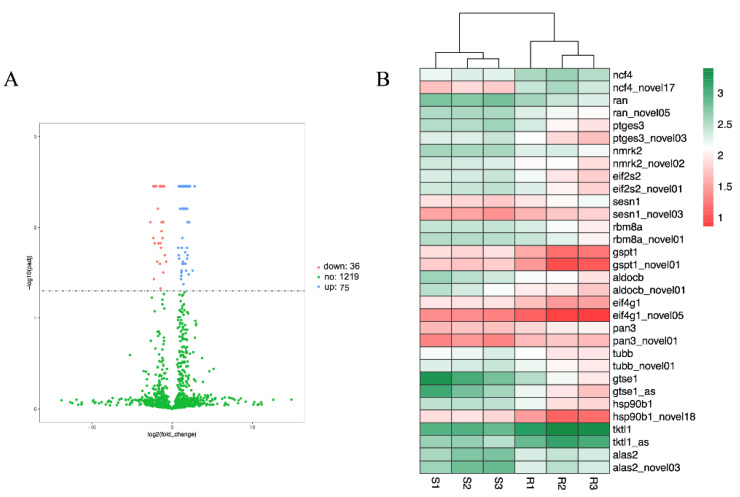
The differentially expressed isoforms between CSBV Bces-Po19-sensitive and -resistant Japanese flounder (*Paralichthys olivaceus*). (**A**) Volcano map of differentially expressed isoforms. Each dot represents one isoform, blue dots represent the significantly up-regulated isoforms, and red dots represent the significantly down-regulated isoforms. Green dots represent isoforms that are not significantly differentially expressed. (**B**) Heatmap analysis of the hierarchical clustering of differentially expressed isoforms with their genes. Different colors indicate differences in the expression level.

Among the 152 genes, 111 isoforms in 92 genes showed different expression patterns between R and S groups, and 75 isoforms were up-regulated in the S group (Figure 10A). Fifteen genes had two differentially expressed isoforms, one gene (GCAT) had three, and the remaining 78 genes had just one differently expressed isoform (Table S10).

### 3.9. Gene Alternative Splicing Is Involved in Signaling Pathways

We investigated the distribution of AS events in enriched signaling pathways. Among the isoforms differentially expressed between R and S groups, 16 isoforms from 16 different genes were involved in 17 signaling pathways (Table S11), including metabolism, immune, and RNA-related process pathways. Five AS isoforms were detected in nine significantly enriched metabolism-related pathways. In addition, five AS isoforms (*hsp90b1_novel18*, *gtse1_as*, *tubb_novel01*, *sesn1_novel03*, and *ncf4_novel17*) were found in three immune-related pathways, namely “phagosome”, “p53 signaling pathway”, and “NOD-like receptor signaling pathway”. Two AS isoforms were found in both the “phagosome” and “p53 signaling pathways”. Six AS isoforms were discovered in the RNA-related pathways: “RNA transport”, “RNA degradation”, “ribosome biogenesis in eukaryotes”, “spliceosome”, and “mRNA surveillance pathway” were found to have six AS isoforms (*pan3_novel01*, *eif4g1_novel05*, *gspt1_novel01*, *rmb8a_novel01*, *eif2s2_novel01*, and *ran_novel05*). The AS isoforms *eif4g1_novel05*, *rmb8a_novel01*, *eif2s2_novel01*, and *ran_novel05* were involved in the “RNA transport” pathway. All the AS isoforms had similar expression patterns with their related genes. A total of twelve isoforms were up-regulated, and four isoforms were down-regulated in the S group in comparison with the R group (Figure 10B).

### 3.10. Identification of TFs

Among the non-redundant unigenes, 1974 were discovered in 60 TF families of the R group, whereas 2179 were found in 58 families of the S group (Table S12). There were 57 TF families shared by the R and S groups. TF families SF-like, TF-box, and Tub were exclusively found in the R group, while HTH was the only TF family found in the S group. Thirteen and ten unigenes were discovered in two TF families in the R and S groups, respectively. Figure S2 shows the top 30 most prevalent TF families in the R and S groups. Members of the zf-C2H2 family were the most numerous, with 527 and 579 members in the R and S groups, respectively, followed by the ZBTB family, with 196 and 255 members, and the third abundant families were TF_bZIP, with 129 members in the R group and bHLH, with 132 members in the S group.

### 3.11. LncRNA Predication

**Figure 11:**
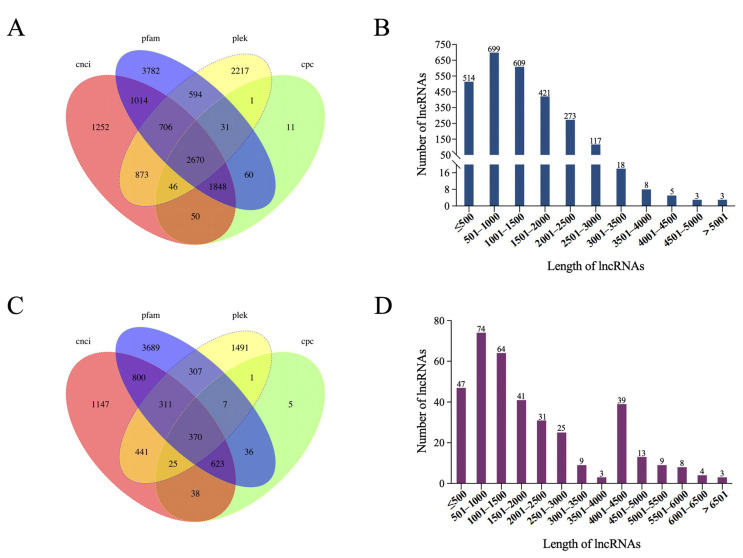
Identification of lncRNAs in CSBV Bces-Po19-sensitive (S) and -resistant (R) Japanese flounder (*Paralichthys olivaceus*). (**A**) Venn diagram of the number of lncRNAs predicted by CNCI, PLEK, CPC, and Pfam in the CSBV Bces-Po19-resistant group. (**B**) Distribution of lncRNAs identified in HCSBV Bces-Po19-resistant group. (**C**) Venn diagram of the number of lncRNAs predicted by CNCI, PLEK, CPC, and Pfam in the CSBV Bces-Po19-sensitive group. (**D**) Distribution of lncRNAs identified in CSBV Bces-Po19-sensitive group.

The number of lncRNA predicted by CNCI, PLEK, CPC, and Pfam from PacBio Iso-Seq data sets was 8459, 7138, 4717, and 10,705 in the R group and 3755, 2953, 1105, and 6143 in the S group, respectively (Figure 11A,C, Table S13). The interaction of these four outcomes produced 2670 lncRNA in the R group and only 370 in the S group, which was significantly less than in the R group. The lengths of the lncRNAs in the R group ranged from 201 to 6267 bp, with an average length of 2565 bp, while lengths in the S group ranged from 216 to 9526 bp, with an average length of 3151 bp (Figure 11B,D). Further research is needed to establish the function of the discovered lncRNAs.

### 3.12. qRT-PCR Validation

The qRT-PCR was performed to confirm the RNA-Seq data. Ten DEGs were chosen randomly selected for PCR amplification, including *rrp1b*, *pycard*, *mthfd2*, *skor1b*, *b3gnt2*, *plcg2*, *vbp1*, *ank2*, *bhmt*, and *rft2*. The subsequent melting curve analysis detected only one product from each primer set. Thus, the qRT-PCR results were consistent with RNA-Seq (Figure S3), demonstrating that RNA-Seq and Trinity assembly were correct.

## 4. Discussion

Fish diseases, notably those triggered by viral infections, have resulted in significant losses in aquaculture. In this study, a novel nidovirus, CSBV Bces-Po19, is reported in marine fish, whereas previous nidoviruses were exclusively found in freshwater fish. The LCDV [14], NNV [31,32], and VHS [33,34] are examples of viruses that may infect both fresh and saltwater fish. The virus’s diverse environmental fitness has made prevention and controls more challenging.

Identifying genes and pathways involved in disease resistance will thus provide the knowledge required for breeding disease-resistant varieties, reducing the economic losses in aquaculture. However, the regulatory mechanisms underlying nidovirus infection and resistance remain unknown. The PacBio Sequel platform was used to analyze the transcript profiles of CSBV Bces-Po19-sensitive and -resistant Japanese flounders in this study. In two libraries, we found a total of 396,000 full-length reads, with an average length of 2550 bp and a maximum length of 14,621 bp. This was significantly longer than the other Japanese flounder transcriptome read lengths obtained using NGS platforms, which were 563 bp [35], 765 bp [36], and 829 bp [10]. Full-length transcripts of high quality are preferred because they provide more data for analyzing the molecular mechanisms involved in CSBV Bces-Po19 infection.

Viral infections trigger a war between the host cells and the viruses to maintain/gain control of vital cell functions critical for the survival of the host cells [37]. One of the primary targets is metabolism, which is a vital set of chemical reactions in the living cells, which also reflect the dynamic expression of genes under specific conditions. It is well-known that virus infections disrupt the metabolism of host cells [38]. Host metabolomic changes are induced during virus infections for various reasons: first, viruses need primary metabolites to synthesize new virions; second, viruses use specially synthesized membranes for viral entry, gene expression, genomic replication, and assembly; and third, signal transduction induced by bioactive lipid metabolites plays a major role in the host response to viral infection and pathogenesis [39]. In this study, 59 metabolism-related signaling pathways were enriched, accounting for 45.94% of the total 129 pathways. The substantial enrichment of metabolism-related pathways suggested that CSBV Bces-Po19 infection triggered metabolic reprogramming in Japanese flounder cells to enable viral genome replication and assembly and thereby viriogenesis. As a result, CSBV Bces-Po19 infection altered the host cell’s metabolic equilibrium, causing a disruption in normal bodily activities and aggravating the host’s sick state.

Teleost fish have evolved innate immunity and adaptive immunity to protect against attack by pathogens [40]. The innate immune system provides the first-line antiviral defense, which is crucial for host defense against invading pathogens [41]. In this study, we discovered that eleven immune-related signaling pathways were enriched in KEGG enrichment analysis. Among these, the phagosome pathway [42], cytosolic DNA-sensing pathways [41], NOD-like receptor signaling pathway [43], Toll-like receptor signaling pathway [44], RIG-I-like receptor signaling pathway [45], lysosome [46], and regulation of autophagy [47] were identified to be involved in multiple innate immune anti-pathogen responses. Further mechanistic studies would be needed to unravel the role of these signaling pathways in antiviral immunity.

Glycolysis is the first step in glucose metabolism in the cells that carry out aerobic and anaerobic respiration. The Warburg effect is an aberrant glycolysis response associated with cancer cells [48]. According to research, the Warburg effect is induced during infection with different viruses such as HCV [49], WSSV [48,50], KSHV [51,52], HSVT-1 [53], DENV [54], EBV [55], and ISKNV [56]. In contrast to previous viruses, Warburg effect-related pathways were not enriched in our investigation, which might be attributed to the diverse sample collection time points. The Warburg effect was identified in CPB cells 24 h after ISKNV infection [56]. The hemocytes in shrimps revealed a strong Warburg effect after 12 h of WSSV infection; however, the Warburg effect did not persist until the late stages of WSSV infection but was found 24 h after infection [48]. The CSBV Bces-Po19 S and R Japanese flounder that we sampled were in late stages of infection, with obvious clinical and pathologic changes. Because glycolysis may not be required by the CSBV Bces-Po19 for survival in host cells at such late stages, the Warburg effect was not detected. More research is needed to determine if CSBV Bces-Po19 may cause the Warburg effect at an early stage of infection.

Aside from metabolism, another confrontation target is AS, which is the splice-site choice that occurs during premature mRNA (pre-mRNA) splicing, resulting in multiple mature mRNAs from a single pre-mRNA. The AS increases transcriptome complexity and thereby the proteome diversity of a cell, and it also modulates the abundance of functional mRNAs [57]. It is common in higher eukaryotes. It is estimated that virtually all genes in humans are alternatively spliced [58]. Virus infections have an impact on the AS events in their host cells as well [37]. In comparison to the control, reticuloendotheliosis virus infection upregulated 2825 AS genes and downregulated 2782 genes in chicken embryo fibroblasts [59]. Infection with the Zika virus in human neural progenitor cells resulted in 262 incidences of AS in 229 genes involved in cell death, RNA processing, transport, and neuron formation [60]. However, whether viral infection can modify the AS landscape in fish has yet to be reported. In this study, we found 12,352 AS events from 10,743 total genes of the CSBV Bces-Po19 S group and 11,452 AS events from 12,989 total genes in the CSBV Bces-Po19 R group. The number and percentage of AS events were greater in the S group than in the R group. Furthermore, among the 152 genes that showed different AS events in the S and R groups, 111 isoforms of 92 genes showed different expression patterns in the R and S groups, and 75 isoforms were up-regulated in the S group, indicating that CSBV Bces-Po19 infection triggered AS events in Japanese flounder. The expression level of the splicing factors affects the outcome of the splicing reaction [61,62,63]. In this study, the splicing factors *sf3a2*, *sf3a3*, *sf3b2*, *sf3b3*, *sf3b4*, *sf3b5*, *u2af1*, *u2af2*, *bcas2,* and *lsy1* were more highly up-regulated in the S group than in the R group. On the one hand, viruses may have driven the up-regulation of splicing factors and changes in AS to aid in their replication, while on the other hand, these processes may have been triggered by the host cell as a self-defense mechanism against the virus.

A variety of viruses have been reported to cause G0/G1, S, or G2/M arrest in the host cells, hence promoting replication of progeny viruses after viral infection [64]. Infection with transmissible gastroenteritis virus, for example, induces cell cycle arrest at S and G2/M phases [65], whereas porcine epidemic diarrhea virus causes cell cycle arrest at the G0/G1 phase [66]. The p53 signaling pathway plays an important role in the regulation of the cell cycle [65,67]. In our study, the p53 signaling pathway was significantly enriched. In addition, one of the genes, *cdk4*/6, necessary for cells to enter the G1 phase from the G0 phase [68], was significantly up-regulated in the CSBV Bces-Po19 S group, indicating that CSBV Bces-Po19 infection might induce G0/G1 phase arrest in the host cells. This is supported by the observed significant enrichment of the cell cycle pathway. Further studies on both the cell cycle arrest and the immune response caused by CSBV Bces-Po19 infection are necessary to confirm the observations reported in this work.

## Figures and Tables

**Table 1:** Summary of full-length transcript sequence data.

	R	S
Polymerase read bases (G)	23.31	24.30
Subreads base (G)	22.43	23.62
CCS	450,095	435,572
NFL	59,771	44,964
FLNC	386,725	383,604
FLNC/CCS	85.92%	88.07%
Polished consensus reads	212,319	183,681
Mean length of polished consensus reads	2186	2922
N50 of polished consensus reads	2643	3429

Note: CCS, circular consensus sequence; NFL, non-full-length non-chimeric read; FLNC, full-length non-chimeric read.

**Table 2:** Summary of enriched immune-related signaling pathways.

Pathways	DEGs Number
p53 signaling pathway	13
Phagosome	17
Cytosolic DNA-sensing pathway	7
NOD-like receptor signaling pathway	7
Salmonella infection	9
Toll-like receptor signaling pathway	7
Herpes simplex infection	11
RIG-I-like receptor signaling pathway	4
Lysosome	8
Apoptosis	4
Regulation of autophagy	1

**Table 3:** Summary of alternative splicing events.

Sample	Total Genes	SE	MX	RI	A5	A3	AF	AL	Total AS
R	12,989	2298 (17.69%)	234 (1.8%)	3018 (23.24%)	2219 (17.08%)	2389 (18.39%)	1049 (8.08%)	245 (1.89%)	11,452
S	10,743	2479 (23.08%)	262 (2.44%)	3358 (31.26%)	2332 (21.71%)	2590 (24.11%)	1120 (10.43%)	211 (1.96%)	12,352

Note: SE, skipped exon; MX, mutually exclusive exon; A5, alternative 5′ splice site; A3, alternative 3′ splice site; RI, retained intron; AF, alternative first exon; AL, alternative last exon; AS, alternative splicing.

**Table 4:** List of genes that difference of alternative splicing number ≥ 10 between S and R.

Gene	AS in S	AS in R	Difference between S and R
*tead3*	27	52	−25
*slco4a1*	57	36	21
*sem4e*	13	34	−21
*mrc1a*	28	9	19
*plcg2*	28	9	19
*cttn*	2	20	−18
*dgki*	12	27	−15
*tktl1*	7	22	−15
*canx*	17	3	14
*sesn1*	4	18	−14
*atp8b2*	27	14	13
*slc29a1*	20	7	13
*mmp25*	4	17	−13
*hnrnpl*	17	6	11
*hspd1*	13	2	11
*ssr1*	9	20	−11
*cpa1*	1	12	−11
*roaa*	15	5	10

## Data Availability

The authors confirm that the data supporting the findings of this study are available within the article and/or its Supplementary Materials.

## Supplementary Materials

The following supporting information can be downloaded at: https://www.mdpi.com/article/10.3390/v14061216/s1, Figure S1: Infection of CSBV Bces-Po19 in cell line EPC, CHSE, and JFB. (A) EPC control. (B) Infected EPC. (C) CHSE control. (D) Infected CHSE. (E) JFB control. (F) Infected JFB. Scale bars: 100 µm; Figure S2: Distribution of the top 30 abundant transcript factors in CSBV Bces-Po19-sensitive and -resistant Japanese flounder (*Paralichthys olivaceus*). (A) Distribution of the top 30 abundant transcript factors in the CSBV Bces-Po19-resistant (R) group. (B) Distribution of the top 30 abundant transcript factors in CSBV Bces-Po19-sensitive (S) group; Figure S3: Validation of the differentially expressed genes by qRT-PCR. The black columns indicate the results of RNA-Seq, and the red columns represent the results of qRT-PCR; TableS1: Primer information used in this study; Table S2: Summary of RNA-seq data; Table S3: The candidate virus that annotated from contigs of small RNA sequence using diseased-fish-tissue-homogenate-inoculated JFB cells; Table S4: Similarity of nucleotides sequences of CSBV Bces-Po19 and other aquatic oncotshaviruses; Table S5: Similarity of amino sequence sequences of CSBV Bces-Po19 and other aquatic oncotshaviruses; Table S6: Length distribution of full-length sequencing reads before and after correction with Illumina RNA-Seq data; Table S7: Summary of mapping rate of full-length sequencing reads to Japanese flounder reference genome; Table S8: Summary of isoform; Table S9: Summary of enriched metabolism-related signaling pathways; Table S10: The differentially expressed isoforms between CSBV Bces-Po19-resistant (R) and -sensitive (S) Japanese flounder (*Paralichthys olivaceus*); Table S11: Gene alternative splicing involved in signal pathways; Table S12: Distribution of transcript factors in CSBV Bces-Po19-resistant (R) and -sensitive (S) Japanese flounder (*Paralichthys olivaceus*); Table S13: Prediction of lncRNA transcripts by PLEK, Pfam, CNCI, and CPC (Y, yes; N, no).
