# Supplementary material for: The Isolation and Full-Length Transcriptome Sequencing of a Novel Nidovirus and Response of Its Infection in Japanese Flounder (Paralichthys olivaceus)
Source: Viruses. 2022 Jun 2;14(6):1216. doi: 10.3390/v14061216 (PMC9230003; doi:10.3390/v14061216)
Supplement: Supplementary file 1 [file viruses-14-01216-s001.zip › Supplementary Files/Table S1.pdf]

Table S1 Primer information used in this study

Gene	Primer sequence (5'-3')	Application
V-ORF1a	F: CGCGCACCACTTAAAACACA<br>R: CGTGAATTGTTGTGCGGGTT	Virus validation
β-actin	F: AGAGCTATGAGCTGCCCGAC<br>R: CTGGGTACATGGTGGTTCCTC	Virus validation
rrp1b	F: AGGGCTTGTGGCGTGATA<br>R: GGATGTGAGTAATCACCGAGT	qRT-PCR
plcg2	F: CCGCTAAAGAACGGCTAC<br>R: CTGGCTGGAGGACGAGTA	qRT-PCR
pycard	F: CAAGACCTCTGACGGAAAG<br>R: CTGTCATTGCCAGGGTCT	qRT-PCR
vbp1	F: TCTCAGGCTGCATCCACA<br>R: GAGACCAGTTCACCACCATC	qRT-PCR
ank2	F: CGATGAAATGCCTGACCTG<br>R: CTTCACGCTCCACGCCAT	qRT-PCR
mthfd2	F: GGGGAGTCTGGGAAATGAT<br>R: GAAATGGTGACCGTGGCA	qRT-PCR
skor1b	F: ATGGCGAGGCAGATGTAG<br>R: GCTCAGCACGGGAAGATA	qRT-PCR
b3gnt2	F: CTGCTGCTGGTGGTCAAGT<br>R: CTGGAGGAGGTCGTTGTGA	qRT-PCR
bhmt	F: GGGCAGACACTCAGTATCAC<br>R: GGGCAGACACTCAGTATCAC	qRT-PCR
rft2	F: GCCTGCTCTAGTGGCTTTA<br>R: GCTGATACTGGGCTTGGA	qRT-PCR
18s	F: GCTGATACTGGGCTTGGA<br>R: AGTTGGTGGAGCGATTTG	qRT-PCR
