# Supplementary material for: The Isolation and Full-Length Transcriptome Sequencing of a Novel Nidovirus and Response of Its Infection in Japanese Flounder (Paralichthys olivaceus)
Source: Viruses. 2022 Jun 2;14(6):1216. doi: 10.3390/v14061216 (PMC9230003; doi:10.3390/v14061216)
Supplement: Supplementary file 1 [file viruses-14-01216-s001.zip › Supplementary Files/Table S11.pdf]

Table S11. Gene alternative splicing involved in signal pathways.

Gene and alternative splicing isoform	log2FoldChange(SvsR) of gene	log2FoldChange(SvsR) of isoform	Pathway
alas2<br>*alas2 _novel03*	1.1161	1.35227	Metabolic pathways
			Porphyrin and chlorophyll metabolism
			Glycine, serine and threonine metabolism
tktl1<br>*tktl1 _as*	-1.2287	-1.45178	Metabolic pathways
			Carbon metabolism
			Biosynthesis of amino acids
hsp90b1<br>*hsp90b1_novel18*	1.4176	2.12447	NOD-like receptor signaling pathway
gtse1<br>*gtse1_as*	2.6997	2.80394	p53 signaling pathway
tubb<br>*tubb_novel01*	0.73377	0.819256	Phagosome
pan3<br>*pan3_novel01*	-0.92075	-1.1412	RNA degradation
eif4g1<br>*eif4g1_novel05*	1.3054	1.43197	RNA transport
aldocb<br>*aldocb_novel01*	1.3487	1.32807	Metabolic pathways
			Carbon metabolism
			Biosynthesis of amino acids
			Pentose phosphate pathway
			Glycolysis / Gluconeogenesis and Fructose and mannose metabolism
gspt1<br>*gspt1_novel01*	1.9074	1.95804	mRNA surveillance pathway
rmb8a<br>*rmb8a_novel01*	0.78439	0.770334	RNA transport
			Spliceosome
			mRNA surveillance pathway
sesn1<br>*sesn1_novel03*	-0.70797	-0.911889	p53 signal pathway
eif2s2<br>*eif2s2_novel01*	1.1146	1.04703	RNA transport
nmrk2<br>*nmrk2_novel02*	0.93177	0.982451	Metabolic pathways
			Nicotinate and nicotinamide metabolism
ptges3<br>*ptges3_novel03*	1.2216	1.25039	Metabolic pathways
			Arachidonic acid metabolism
ran<br>*ran_novel05*	1.1096	1.1465	RNA transport
			Ribosome biogenesis in eukaryotes
ncf4	-0.98717	-2.17532	Phagosome

ncf4_novel17			
