# Supplementary material for: The Isolation and Full-Length Transcriptome Sequencing of a Novel Nidovirus and Response of Its Infection in Japanese Flounder (Paralichthys olivaceus)
Source: Viruses. 2022 Jun 2;14(6):1216. doi: 10.3390/v14061216 (PMC9230003; doi:10.3390/v14061216)
Supplement: Supplementary file 1 [file viruses-14-01216-s001.zip › Supplementary Files/Table S2.pdf]

Table S2. Summary of RNA-seq data.

Sample	Total clean reads	Total mapping rate	FPKM Interval			
			1-5	5-15	15-60	>60
R1	41175958	88.71%	3485(14.32%)	4293(17.64%)	4384(18.02%)	1645(6.76%)
R2	55451184	89.74%	3593(14.76%)	4406(18.11%)	4251(17.47%)	1512(6.21%)
R3	53561556	89.13%	3779(15.53%)	4915(20.20%)	4914(20.19%)	1445(5.94%)
S1	45660498	90.95%	3134(12.88%)	4222(17.35%)	4468(18.36%)	1681(6.91%)
S2	51103116	91.40%	3115(12.80%)	4242(17.43%)	4509(18.53%)	1679(6.90%)
S3	55892426	90.83%	3146(12.93%)	4208(17.29%)	4472(18.38%)	1736(7.13%)
