# Supplementary material for: The Isolation and Full-Length Transcriptome Sequencing of a Novel Nidovirus and Response of Its Infection in Japanese Flounder (Paralichthys olivaceus)
Source: Viruses. 2022 Jun 2;14(6):1216. doi: 10.3390/v14061216 (PMC9230003; doi:10.3390/v14061216)
Supplement: Supplementary file 1 [file viruses-14-01216-s001.zip › Supplementary Files/Table S3.pdf]

Table S3. The candidate virus that annotated from contigs of small RNA sequence using diseased fish tissue homogenate inoculated JFB cells.

| Database | Virus name | Annotated contigs number | Annotated contigs length | Hit length | Annotated contigs id |
|---|---|---|---|---|---|
| NR | Chinook salmon bafinivirus | 1 | 247 | 79 | NODE_9_length_247_cov_0.352679 |
| NT | MG600027.1 Chinook salmon bafinivirus strain WHQSR4345 1ab protein, spike protein, membrane protein, and nucleoprotein genes, complete cds | 2 | 595 | 577 | NODE_8_length_348_cov_1.378462, NODE_9_length_247_cov_0.352679 |
| NT | MT123520.1 Chinook salmon bafinivirus isolate CefasW054, complete genome | 1 | 184 | 72 | NODE_15_length_184_cov_0.130435 |
| Virus RefSeq Nucletide | Proteus phage vB_PmiS-TH | 1 | 283 | 247 | Contig8 |
| Virus RefSeq Nucletide | Chinook salmon bafinivirus isolate NIDO | 3 | 721 | 444 | NODE_15_length_184_cov_0.130435, NODE_8_length_348_cov_1.378462, NODE_12_length_189_cov_1.536145 |
| Virus RefSeq Nucletide | Shamonda virus N and NSs genes | 2 | 2992 | 182 | NODE_2_length_1194_cov_46.914603, Contig4 |
| Virus RefSeq Nucletide | Choristoneura occidentalis granulovirus | 5 | 10626 | 458 | Contig10, Contig3, Locus_1_Transcript_12/25_Confidence_0.185_Length_2533, Contig2, Contig1 |
| Virus RefSeq Protein | Chinook salmon bafinivirus | 2 | 431 | 103 | NODE_9_length_247_cov_0.352679, NODE_15_length_184_cov_0.130435 |
