# Supplementary material for: The Isolation and Full-Length Transcriptome Sequencing of a Novel Nidovirus and Response of Its Infection in Japanese Flounder (Paralichthys olivaceus)
Source: Viruses. 2022 Jun 2;14(6):1216. doi: 10.3390/v14061216 (PMC9230003; doi:10.3390/v14061216)
Supplement: Supplementary file 1 [file viruses-14-01216-s001.zip › Supplementary Files/Table S4.pdf]

Table S4. Similarity of nucleotides sequences of CSBV Bces-Po19 and other aquatic oncotshaviruses.

Virus strain (species)	Genome size (nt)	Nucleotide sequence identity (%)					
		Whole genome sequences	ORF 1a	ORF 1b	S	M	N
CSBV WHQSR4345	26466	98.62	99.50	97.96	99.06	98.51	99.25
CSBV HB93	25971	96.13	85.71	93.44	98.95	99.19	99.44
CSBV Cefas-W054	25969	95.21	94.33	97.30	98.26	97.43	86.59
CSBV NIDO	27004	94.69	93.24	97.59	97.71	96.88	86.03
ASBV	26496	96.65	96.94	97.44	98.23	98.37	86.03
YCBV	26985	93.17	91.50	96.13	94.69	85.13	97.19
