# Supplementary material for: The Isolation and Full-Length Transcriptome Sequencing of a Novel Nidovirus and Response of Its Infection in Japanese Flounder (Paralichthys olivaceus)
Source: Viruses. 2022 Jun 2;14(6):1216. doi: 10.3390/v14061216 (PMC9230003; doi:10.3390/v14061216)
Supplement: Supplementary file 1 [file viruses-14-01216-s001.zip › Supplementary Files/Table S5.pdf]

Table S5. Similarity of amino sequence sequences of CSBV-Bces-Po19 and other aquatic oncotshaviruses.

Virus strain (species)	Amino acid sequence identity (%)				
	ORF 1a	ORF 1b	S	M	N
CSBV WHQSR4345	99.49	99.21	98.51	98.78	99.44
CSBV HB93	85.99	94.34	98.42	98.78	100.00
CSBV Cefas-W054	94.80	98.77	97.76	97.55	85.39
CSBV NIDO	93.85	99.04	97.10	96.33	84.27
ASBV	97.56	99.04	97.43	99.18	84.27
YCBV	91.75	98.64	93.53	84.84	97.74
