# Supplementary material for: The Isolation and Full-Length Transcriptome Sequencing of a Novel Nidovirus and Response of Its Infection in Japanese Flounder (Paralichthys olivaceus)
Source: Viruses. 2022 Jun 2;14(6):1216. doi: 10.3390/v14061216 (PMC9230003; doi:10.3390/v14061216)
Supplement: Supplementary file 1 [file viruses-14-01216-s001.zip › Supplementary Files/Table S6.pdf]

Table S6. Length distribution of full-length sequencing reads before and after correction with Illumina RNA-Seq data.

Sample	Type	Total nucleotides	Total number	Mean length	Min length	Max length	N50	N90
R	Before correct	464072319	212319	2186	198	13823	2643	1399
	After correct	463495705	212319	2184	182	12987	2641	1398
S	Before correct	536642204	183681	2922	181	14634	3429	1888
	After correct	535777939	183681	2917	182	14621	3420	1887
