# Supplementary material for: The Isolation and Full-Length Transcriptome Sequencing of a Novel Nidovirus and Response of Its Infection in Japanese Flounder (Paralichthys olivaceus)
Source: Viruses. 2022 Jun 2;14(6):1216. doi: 10.3390/v14061216 (PMC9230003; doi:10.3390/v14061216)
Supplement: Supplementary file 1 [file viruses-14-01216-s001.zip › Supplementary Files/Table S7.pdf]

Table S7. Summary of mapping rate of full-length sequencing reads to Japanese flounder reference genome.

Sample	Total reads	Total mapped	Unmapped	Multiple mapped	Uniquely mapped	Reads map to '+'	Reads map to '-'
R	212319	184371 (86.84%)	27948 (13.16%)	2938 (1.38%)	181433 (85.45%)	100678 (47.42%)	80755 (38.03%)
S	183681	155400 (84.6%)	28281 (15.4%)	1578 (0.86%)	153822 (83.74%)	81780(44.52%)	72042 (39.22%)
