# Supplementary material for: The Isolation and Full-Length Transcriptome Sequencing of a Novel Nidovirus and Response of Its Infection in Japanese Flounder (Paralichthys olivaceus)
Source: Viruses. 2022 Jun 2;14(6):1216. doi: 10.3390/v14061216 (PMC9230003; doi:10.3390/v14061216)
Supplement: Supplementary file 1 [file viruses-14-01216-s001.zip › Supplementary Files/Table S8.pdf]

Table S8. Summary of isoform.

	R	S
Isoform number	45901	42177
Isoforms of known genes	2641	2502
Novel isoform of known genes	40423	38716
Isoforms of novel genes	2837	959
Isoform mean length	2565.32	3151.50
N50	2981	3711
