# Supplementary material for: The Isolation and Full-Length Transcriptome Sequencing of a Novel Nidovirus and Response of Its Infection in Japanese Flounder (Paralichthys olivaceus)
Source: Viruses. 2022 Jun 2;14(6):1216. doi: 10.3390/v14061216 (PMC9230003; doi:10.3390/v14061216)
Supplement: Supplementary file 1 [file viruses-14-01216-s001.zip › Supplementary Files/Table S9 .pdf]

Table S9. Summary of enriched metabolism related signaling pathways.

Pathways	DEGs number
Metabolic pathways	144
Pyrimidine metabolism	26
Purine metabolism	31
Oxidative phosphorylation	22
Glutathione metabolism	11
Carbon metabolism	17
Propanoate metabolism	7
Cysteine and methionine metabolism	8
Drug metabolism - other enzymes	7
Glyoxylate and dicarboxylate metabolism	6
Pyruvate metabolism	7
Fatty acid metabolism	8
Porphyrin and chlorophyll metabolism	6
Citrate cycle (TCA cycle)	6
Biosynthesis of amino acids	10
Valine, leucine and isoleucine degradation	7
Fatty acid biosynthesis	4
One carbon pool by folate	4
Fatty acid degradation	6
Synthesis and degradation of ketone bodies	2
2-Oxocarboxylic acid metabolism	4
Glycine, serine and threonine metabolism	6
Arachidonic acid metabolism	6
Selenocompound metabolism	3
Alanine, aspartate and glutamate metabolism	5
Glycerolipid metabolism	6
Pentose phosphate pathway	4
beta-Alanine metabolism	4
Tryptophan metabolism	5
Glycerophospholipid metabolism	8
Riboflavin metabolism	2
Sulfur metabolism	2
Butanoate metabolism	3
Ether lipid metabolism	4
Inositol phosphate metabolism	6
Folate biosynthesis	2
Arginine and proline metabolism	6
Primary bile acid biosynthesis	2
Terpenoid backbone biosynthesis	2
Nicotinate and nicotinamide metabolism	3

Nitrogen metabolism	2
Tyrosine metabolism	2
Amino sugar and nucleotide sugar metabolism	3
Retinol metabolism	2
Fructose and mannose metabolism	2
Linoleic acid metabolism	1
Sphingolipid metabolism	2
Drug metabolism - cytochrome P450	1
Starch and sucrose metabolism	1
Glycosaminoglycan degradation	2
Lysine degradation	4
Glycosphingolipid biosynthesis - lacto and neolacto series	2
Glycolysis / Gluconeogenesis	4
Fatty acid elongation	2
Pantothenate and CoA biosynthesis	1
Biosynthesis of unsaturated fatty acids	1
N-Glycan biosynthesis	2
Mucin type O-Glycan biosynthesis	1
Other types of O-glycan biosynthesis	1
