# Supplementary figures and images for: The Isolation and Full-Length Transcriptome Sequencing of a Novel Nidovirus and Response of Its Infection in Japanese Flounder (Paralichthys olivaceus)
Source: Viruses. 2022 Jun 2;14(6):1216. doi: 10.3390/v14061216 (PMC9230003; doi:10.3390/v14061216)

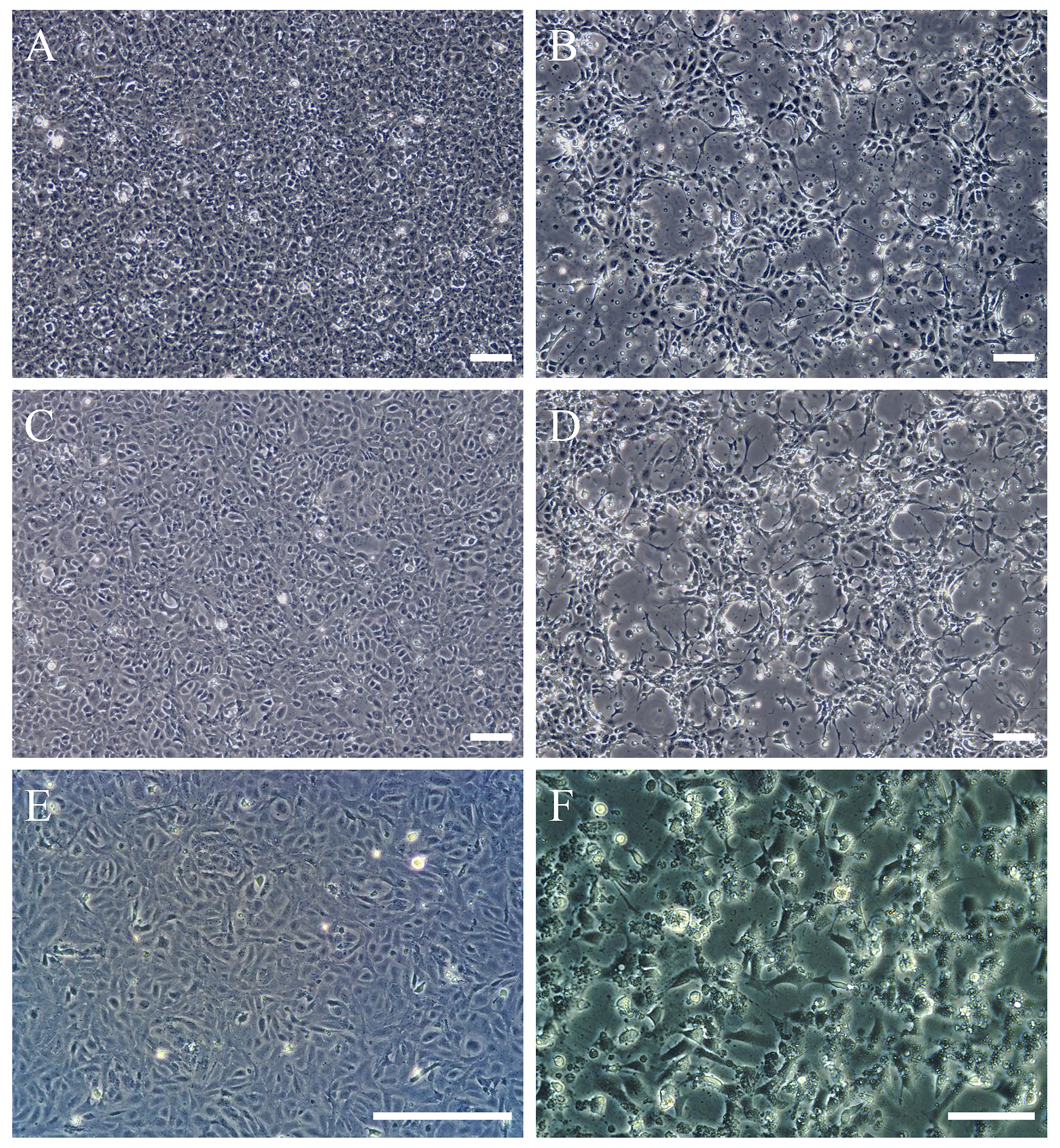

Supplement: Supplementary file 1 [file viruses-14-01216-s001.zip › Supplementary Files/Figure S1.tif]

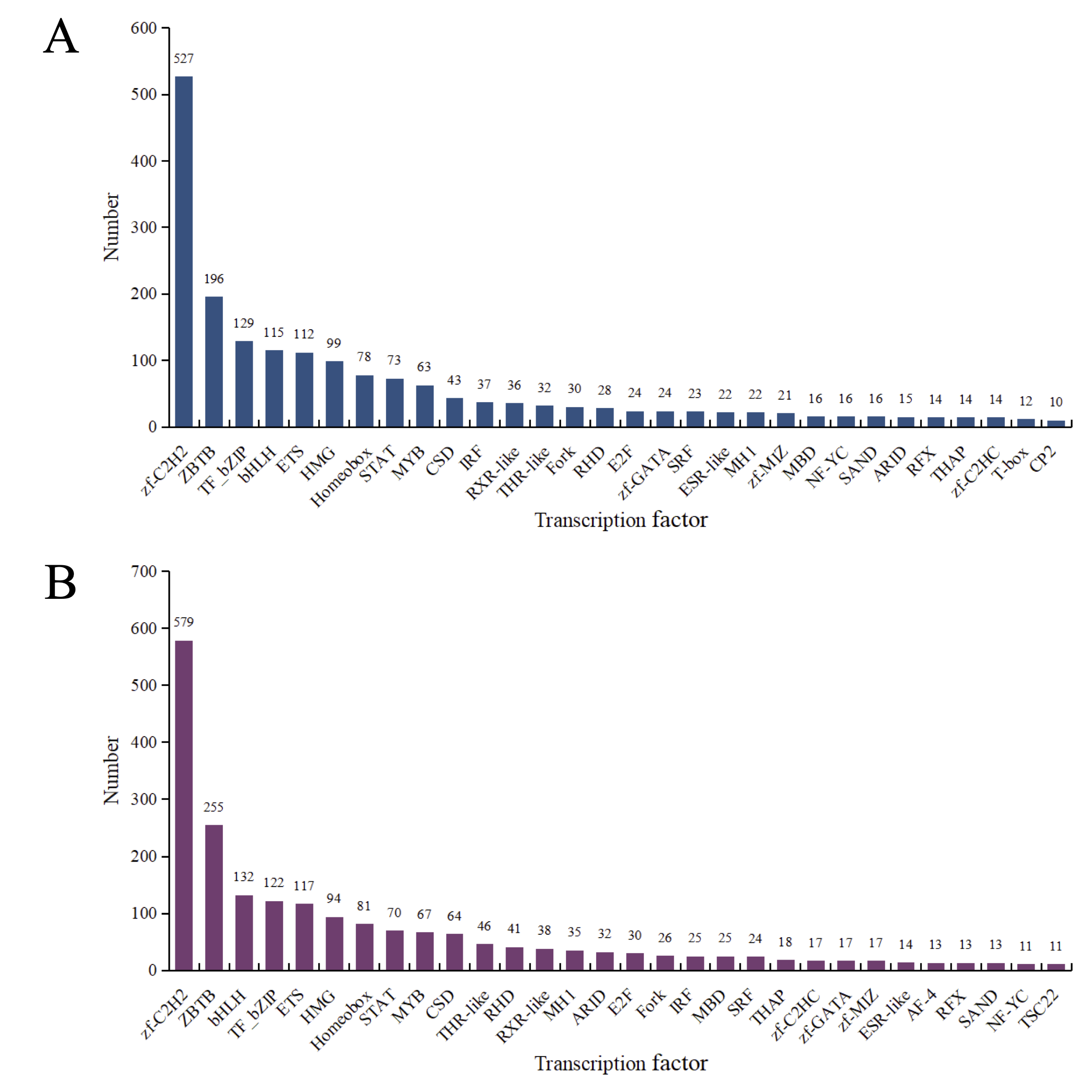

Supplement: Supplementary file 1 [file viruses-14-01216-s001.zip › Supplementary Files/Figure S2.tiff]

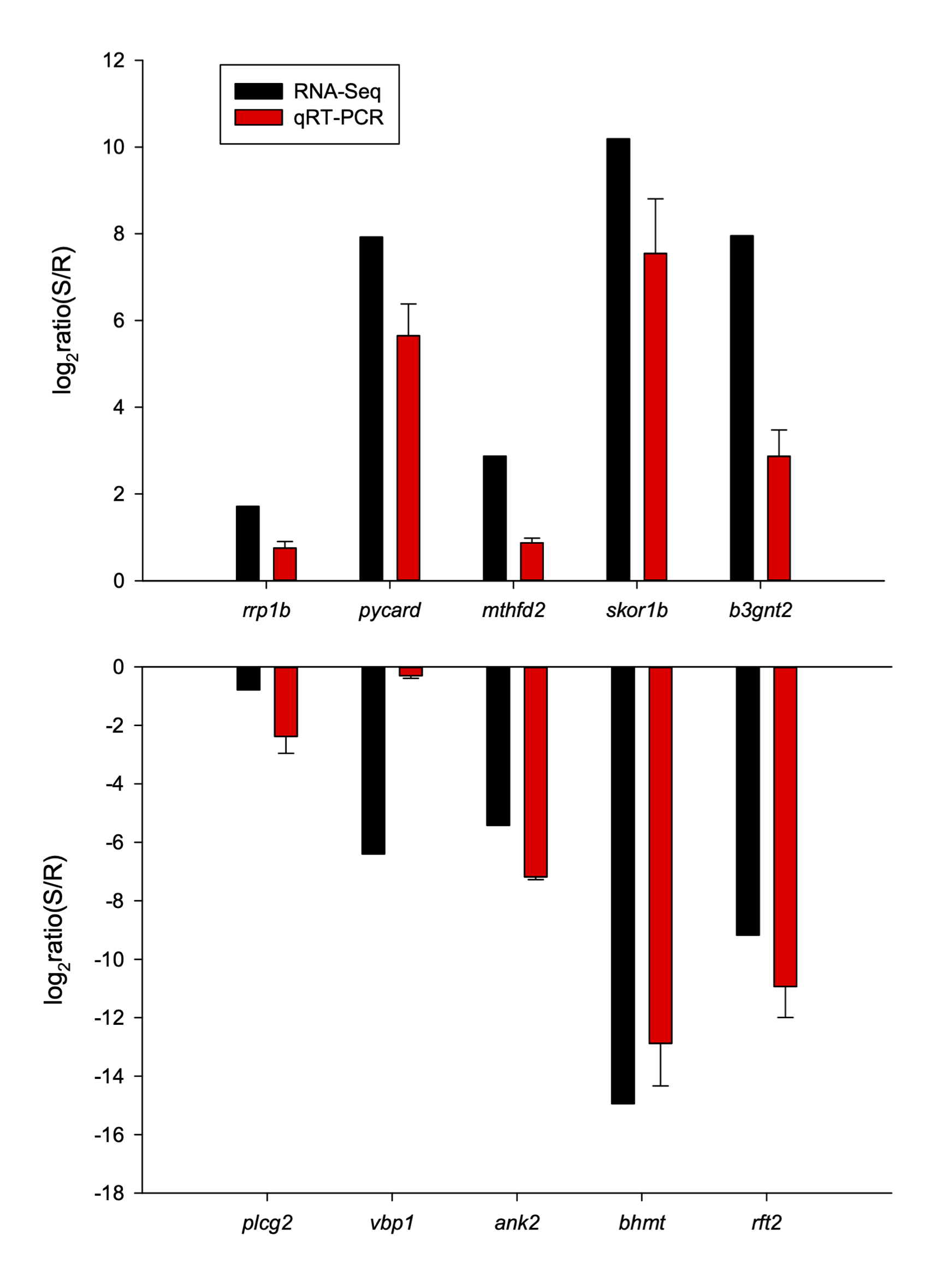

Supplement: Supplementary file 1 [file viruses-14-01216-s001.zip › Supplementary Files/Figure S3.tiff]
